# Photonic Dipstick Immunosensor to Detect Adulteration of Ewe, Goat, and Donkey Milk with Cow Milk through Bovine κ-Casein Detection

**DOI:** 10.3390/s24175688

**Published:** 2024-08-31

**Authors:** Dimitra Kourti, Michailia Angelopoulou, Eleni Makarona, Anastasios Economou, Panagiota Petrou, Konstantinos Misiakos, Sotirios Kakabakos

**Affiliations:** 1 Immunoassays–Immunosensors Lab, Institute of Nuclear & Radiological Sciences & Technology, Energy & Safety, NCSR “Demokritos”, GR-15341 Aghia Paraskevi, Greece; d.kourti@rrp.demokritos.gr (D.K.); mikangel@ipta.demokritos.gr (M.A.); ypetrou@rrp.demokritos.gr (P.P.); 2 Analytical Chemistry Lab, Department of Chemistry, National and Kapodistrian University of Athens, Panepistimiopolis Zografou, GR-15771 Athens, Greece; aeconomo@chem.uoa.gr; 3 Institute of Nanoscience & Nanotechnology, NCSR “Demokritos”, GR-15341 Aghia Paraskevi, Greece; e.makarona@inn.demokritos.gr (E.M.); k.misiakos@inn.demokritos.gr (K.M.)

**Keywords:** photonic chip immunosensor, Mach–Zehnder interferometry, label-free detection, milk adulteration, bovine κ-casein

## Abstract

The quality and authenticity of milk are of paramount importance. Cow milk is more allergenic and less nutritious than ewe, goat, or donkey milk, which are often adulterated with cow milk due to their seasonal availability and higher prices. In this work, a silicon photonic dipstick sensor accommodating two U-shaped Mach–Zehnder Interferometers (MZIs) was employed for the label-free detection of the adulteration of ewe, goat, and donkey milk with cow milk. One of the two MZIs of the chip was modified with bovine κ-casein, while the other was modified with bovine serum albumin to serve as a blank. All assay steps were performed by immersion of the chip side where the MZIs are positioned into the reagent solutions, leading to a photonic dipstick immunosensor. Thus, the chip was first immersed in a mixture of milk with anti-bovine κ-casein antibody and then in a secondary antibody solution for signal enhancement. A limit of detection of 0.05% *v*/*v* cow milk in ewe, goat, or donkey milk was achieved in 12 min using a 50-times diluted sample. This fast, sensitive, and simple assay, without the need for sample pre-processing, microfluidics, or pumps, makes the developed sensor ideal for the detection of milk adulteration at the point of need.

## 1. Introduction

Milk and dairy products have high nutritional value, and their consumption contributes to a balanced and nutritious diet. As milk production and consumption have increased over the years, milk adulteration incidents are also more frequent [1,2,3,4,5]. Milk adulteration includes the addition of vegetable protein, the mixing of milk from different species, and the addition of whey and/or water. This type of adulteration is primarily driven by the pursuit of financial gain, especially when milk from species other than cows is considered, such as ewe, goat, and especially donkey milk [6,7]. Adulteration of milk from these species with cow milk is not, however, only a matter of financial fraud but could cause severe health implications when it is consumed by individuals who are sensitive or allergic to cow milk. As a result, milk adulteration poses a significant threat to public health. In response, the European Commission (EC) has established 1% *v*/*v* as the maximum acceptable limit of cow milk content in dairy products from other species [8].

To detect milk adulteration, several methods have been developed, such as chromatographic, spectrometric, molecular, immunological, and biosensing ones [2,3,4]. Different chromatographic techniques have been used in order to detect traces of cow milk in milk from other species [9,10,11]. For example, high-performance liquid chromatography coupled with electrospray ionization mass-spectrometry has been used to detect the fraudulent addition of cow milk in camel milk powder [9] and goat milk [10]. In addition, a gas chromatography–mass spectrometry-based metabolomic method that combined chromatography with multivariate statistical data analysis was applied to detect the adulteration of goat with cow milk based on the differences in their polar metabolite profiles [12]. Other methods, such as Polyacrylamide Gel Electrophoresis (PAGE) [13] and MALDI-TOF-MS [10], have been also employed for the detection of milk adulteration. The PAGE method was based on the detection of bovine b-lactoglobulins in caprine/bovine and ovine/bovine milk mixtures and bovine a-lactalbumin in caprine/bovine milk [13]. On the other hand, MALDI-TOF-MS enabled the detection of cow and goat milk in the milk of other species through evaluation of species-specific markers in the peptide profiles of tryptic digests of the respective milks [14,15,16]. All these methods had detection limits in the range of 3 to 5% and required expensive instrumentation and trained personnel.

Polymerase chain reaction (PCR)-based methods have also been developed for the detection of cow DNA in samples of goat, ewe, and buffalo milk [17,18,19]. Although these methods provide high sensitivity and specificity, they require DNA extraction from the milk samples prior to analysis and therefore are not suited for on-site determinations. In another report, a loop-mediated isothermal amplification (LAMP) method was combined with a lateral flow strip to create a portable platform for on-site detection of goat milk adulteration with cow milk in about 50 min with a detection limit of 5% *v*/*v* [20].

Immunological methods, including enzyme-linked immunosorbent assays (ELISA) and lateral flow immunoassays, have also been developed for the detection of milk adulteration [21]. More specifically, ELISAs for the accurate detection of cow milk in ewe milk and cheese [22] or yak milk [23] have been reported in the literature. ELISA is widely used in the food industry due to its high sensitivity and specificity; however, it is a time-consuming laboratory-bound method. Adulterated goat milk products have also been successfully detected by a lateral flow immunoassay (LFIA), which is suitable for on-site determinations but provides semi-quantitative results [24].

To overcome these limitations, biosensing platforms based on different transducer principles, mostly electrochemical [25], optical [26], and piezoelectric [27], are gaining ground in the food analysis sector since they could provide fast quantitative analysis at the point of need. Thus, a quartz crystal microbalance (QCM) immunosensor was developed for the detection of adulteration with cow milk through the measurement of a 208 kDa protein that is found in cow milk but not in goat [28]. However, only detection in buffer and not in milk samples was demonstrated. Voltametric and amperometric electrochemical sensors, both based on the detection of bovine γ-globulins, have shown adequate sensitivity (0.1% *v*/*v*) in terms of cow milk detection in the milk of other species, with an assay duration from 0.5 to 1 h [29,30]. Furthermore, an electronic tongue with 36 cross-sensibility potentiometric sensors was evaluated for the determination of adulterated milk and was able to distinguish between goat, cow, and goat/cow raw skimmed milk, based on the different signal profiles of each milk sample [31]. Despite this, in order to be used for routine analysis by the dairy industry, this electronic tongue system must be improved through the inclusion of more sensors sensible to milk composition variations. Regarding optical biosensors, a benchtop [32] and a portable surface plasmon resonance sensor [33] have been applied to detect goat and/or ewe milk adulteration with cow milk at detection limits of 0.1 and 0.17% *v*/*v* with assay times of 5 and 7 min, respectively.

Our team has developed optical immunosensors that are fully integrated onto silicon chips in order to detect various harmful food contaminants, such as toxins and allergens [34,35,36,37]. In this context, integrated Mach–Zehnder interferometers (MZIs) have been employed for the precise determination of bovine milk in goat milk with a limit of detection of 0.1% *v*/*v*, which is achievable with a 10 min assay [34]. The same approach was applied for the quantification of bovine milk in protected designation of origin (PDO) cheeses, namely mozzarella and feta, at concentrations as low as 0.5% and 0.25% *w*/*w*, respectively [36].

In this study, a novel chip design is exploited for the quantitative detection of cow milk in adulterated ewe, goat, and donkey milk through the immunochemical determination of bovine κ-casein. The chip incorporates two U-shaped silicon nitride waveguides with light input and output onto the same chip side [38]. On the other side, the waveguides have been formatted as MZIs, enabling bioreactions to be monitored by immersion of this end into immunoassay reagents. This way, there was no need for the use of microfluidics and pumps. The biosensing platform is completed by a broad-band white light source and a spectrophotometer, both coupled to the photonic dipstick sensor via a bifurcated optical fiber. Connection between the chip and the bifurcated fiber was achieved by means of a custom-designed adapter that ensures the alignment of the on-chip waveguide inputs and outputs to the branches of the bifurcated fiber. The whole system is powered up by the laptop that also runs the software for signal recording and processing. The presence of two sensors per chip provides the ability to use one of them to monitor the specific immunoreaction, and the other one to monitor refractive index changes due to non-specific binding or sample matrix effects. The MZI-based immersible immunosensor used in this work has already been employed in preliminary experiments to detect bovine κ-casein added to an assay buffer and goat or ewe milk [39]. In this work, the detection of goat, ewe, and donkey milk adulteration with cow milk is demonstrated. For this purpose, the MZI with the longer sensing arm opening was modified with bovine κ-casein and the sensing arm of the other ΜΖΙ with bovine serum albumin. A rabbit polyclonal antibody against bovine κ-casein was implemented to develop a competitive immunoassay for the determination of cow’s milk in ewe, goat, and donkey milk. The primary immunoreaction was followed by a reaction with a goat antibody against rabbit γ-globulins (secondary antibody) to enhance the specific signal and reduce the assay duration. The effect of each milk matrix on the assay was investigated and the assay parameters were optimized so as to obtain the highest possible sensitivity for the shortest assay duration and to take full advantage of the biosensing system portability for milk adulteration detection at the point of need.

## 2. Materials and Methods

### 2.1. Reagents

Bovine κ-casein, goat anti-rabbit IgG antibody (secondary antibody), and 3-aminopropyltriethoxysilane (APTES) were purchased from Sigma-Aldrich Co. (Saint Louis, MO, USA). Bovine serum albumin (BSA) was obtained from Acros Organics BV (Geel, Belgium). The anti-bovine κ-casein rabbit antiserum was developed in-house as previously described [36]. All other chemicals and reagents were obtained from Merck KGaA (Darmstadt, Germany). The water used throughout the study was doubly distilled.

### 2.2. Milk Samples

Pasteurized cow (3.5% fat; DELTA FOODS S.A.; Agios Stefanos, Attiki, Greece), ewe (6.0% fat; Omada Paragogon “Gianniotiko” I.K.E; Neochori, Ioannina, Greece), goat (3.5% fat; Larisa Dairy S.A. “OLYMPUS”; Larisa, Greece), and donkey milk (0.6% fat; Farma Metsovou; Chrisovitsa, Ioannina, Greece) were purchased from the local market. For milk preservation, sodium azide was added at a final concentration of 0.05% *w*/*v*, and the milk samples were then aliquoted and stored at −20 °C.

### 2.3. Photonic Silicon Chip Fabrication, Principle of Operation, and Instrumentation

**Figure 1:**
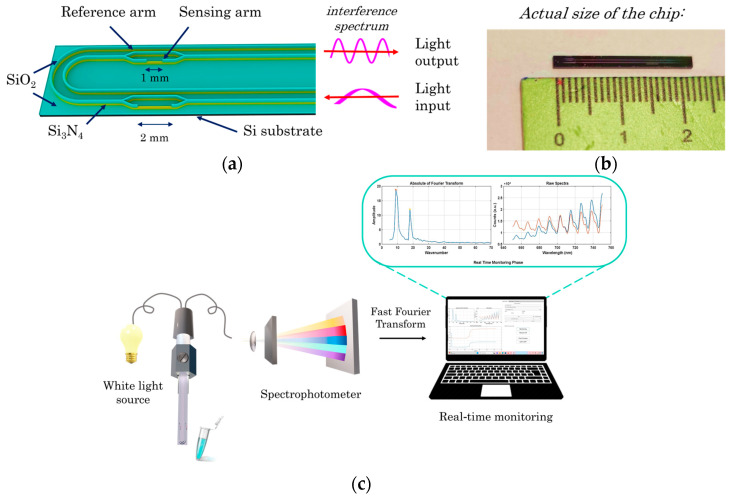
(**a**) Schematic of the chip layout, (**b**) actual size of the chip, and (**c**) schematic of the optical set-up and signal processing software.

The integrated photonic silicon chips have been fabricated in the clean room facility of the Institute of Nanoscience and Nanotechnology of NCSR “Demokritos”, as has been described previously [38]. The waveguides were defined by e-beam lithography (Raith EBPG 5000plusES; Raith GmbH, Dortmund, Germany) and subsequent reactive ion etching (Alcatel Nextral NE330) on 4” silicon wafers with thermally grown 5 μm thick thermal SiO_2_ and 150 nm Si_3_N_4_ deposited on top of the oxide (Si-mat Silicon Materials, Kaufering, Germany), as schematically depicted in Figure S1. The oxide layer played the role of the waveguide undercladding layer, while the nitride layer was the waveguide core. The waveguides at the Y junctions and the coupling edges were 250 nm wide to ensure monomodality in the red spectral region and to maximize coupling efficiency to the bi-furcated fiber through the optomechanical coupler. On top of the etched waveguides, a 2 μm LPCVD SiO_2_ layer was deposited (Tempress Omega Junior; Vaasen, The Netherlands) to serve as the overcladding of the waveguides. Finally, optical lithography (SÜSS MicroTec MA6/BA6; SÜSS MicroTec SE, Garching, Germany) followed by standard silicon oxide wet etching was employed to define the sensing windows. Both sensing windows had a width of 20 μm, but their lengths were 1 and 2 mm, respectively (Figure 1a). After dicing, chips with dimensions of 22 mm × 2.0 mm were obtained (Figure 1b).

For the real-time monitoring of refractive index changes over the sensing windows of the two MZIs, the chips were connected to a broad-band high-brightness white light source (Ushio Europe B.V.; Oude Meer, The Netherlands) and a VIS-NIR spectrophotometer (Flame-T-VIS-NIR, Ocean Insight; Duiven, The Netherlands) through a 200 μm core bifurcated optical fiber (Ocean Insight; Duiven, The Netherlands) (Figure 1c). The alignment of the chip light input and output with the bifurcated optical fiber is realized using a specially designed optical adapter fabricated by 3D printing (Figure S2). The spectrophotometer is connected to a laptop that also runs the software for signal acquisition and data analysis (Figure S2).

The combined transmission spectrum of both MZIs is continuously recorded throughout the assay. The different opening lengths of the two MZIs result in peaks corresponding to two different frequencies in the respective interferometric spectrum. After applying the Fast Fourier Transform, the wavenumbers of the two spectra are determined, allowing the response of each MZI to be monitored separately in real-time as a phase shift due to interactions taking place on them (Figure S3).

### 2.4. Chemical and Biological Functionalization of the Photonic Chip

The photonic silicon chips were chemically activated prior to the deposition of bovine κ-casein and BSA in the sensing arm windows of the two MZIs. This is performed through hydrophilization by Piranha treatment of the chips, followed by aminosilanization. More specifically, the chips were immersed in Piranha solution (1:1 volume mixture of H_2_SO_4_ and 30% *v*/*v* H_2_O_2_) for 20 min, washed with distilled H_2_O, and dried under a nitrogen stream. The dried chips were then immersed in a 2% *v*/*v* APTES solution in absolute ethanol for 20 min, washed with absolute ethanol, dried with nitrogen, and cured at 120 °C for 20 min. The chips’ biological activation was performed using a microarray spotter (BioOdyssey Calligrapher Mini Arrayer; (Bio-Rad Laboratories Inc.; Hercules, CA, USA) by applying overlapping spots using a solid pin with a 375 μm diameter (Arrayit Corp.; Sunnyvale, CA, USA). The MZI with the 2 mm sensing arm opening (working sensor) is modified with a 50 μg/mL bovine κ-casein solution in 50 mM carbonate buffer, pH 9.2, and the sensing arm opening of the other MZI (reference MZI) with a 50 μg/mL BSA solution in the same buffer. Then, the chips were incubated for 1 h in a humidity chamber (approx. 70% humidity), washed with distilled water, dried with nitrogen, and kept in a desiccator at RT until use.

### 2.5. Assay for Detection of Bovine Milk in Milk from Other Species with the Photonic Dipstick Immunosensor

For the assay, the chemically modified and biofunctionalized chips, coupled to the optical fiber, were immersed for 2 min in microtiter wells containing 300 μL of equilibration buffer (0.01 M PBS, pH 7.4, 5 g/L BSA, 0.5 g/L Tween^®^ 20, 1% *v*/*v* ewe or goat or donkey milk) for chip signal equilibration. Then, the chips were immersed in 300 μL of a 1:1 volume mixture of calibrators or 50-times diluted milk samples with anti-bovine κ-casein antiserum 200-times diluted with assay buffer (0.01 M PBS, pH 7.4, 5 g/L BSA, 0.5 g/L Tween^®^ 20) for 5 min. After that, the chips were washed by immersion for 2 min in equilibration buffer, followed by submersion in 300 μL of a 10 μg/mL goat anti-rabbit IgG-specific antibody solution prepared in equilibration buffer for 5 min. Finally, the chips were regenerated by immersion in 300 μL of 50 mM HCl solution to remove the antibodies bound on the sensors and then in equilibration buffer prior to the next run. All assay and washing steps were performed under stirring (400 rpm). For the calibration curve, the net chip signal corresponding to each calibrator was calculated by applying the following equation:

S_x_ = (Working sensor phase shift) – 2 × (Reference sensor phase shift)

**Figure 2:**
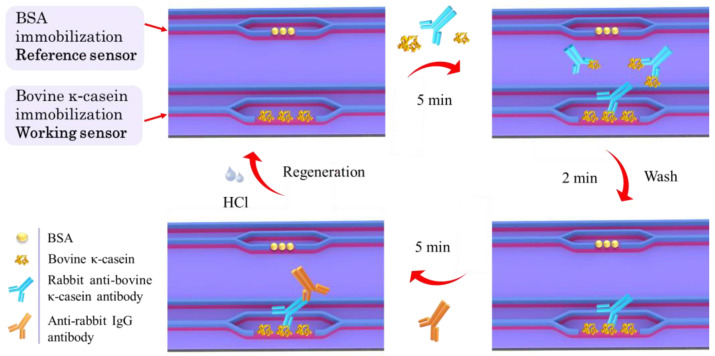
Schematic of the competitive immunoassay steps for detection of cow milk in milk from other species with the photonic dipstick sensor.

The correction of the working sensor signal with respect to that of the reference one also resulted in the smoothing out of “peaks” observed during real-time signal monitoring due to the movement of the chip from one reagent well to another (lower panel in Figure S3a–c). Then, the percent ratio of each calibrator net signal (S_x_) to the zero calibrator net signal (S_0_) was plotted against the percent ratio of cow milk in ewe, goat, or donkey milk in the respective calibrators. A schematic of the competitive immunoassay steps is depicted in Figure 2.

## 3. Results and Discussion

### 3.1. Chemical Modification of the Chip

**Figure 3:**
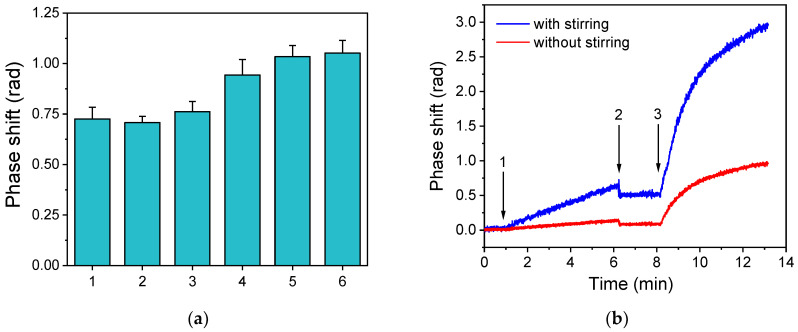
(**a**) Net zero calibrator signals obtained with chips modified with APTES following different protocols: 0.5% *v*/*v* APTES in H_2_O, 2 min (1); 2% *v*/*v* APTES in H_2_O, 2 min (2); 2% *v*/*v* APTES in H_2_O, 20 min (3); 2% *v*/*v* APTES in ethanol, 2 min (4); 2% *v*/*v* APTES in ethanol, 20 min (5); and 2% *v*/*v* APTES in ethanol, 60 min. All chips have been spotted with a 100 μg/mL bovine κ-casein solution and assayed with a 50-times diluted anti-bovine κ-casein rabbit antiserum. Each column corresponds to the mean of 3 chips ± SD. (**b**) Real-time signal responses obtained for a κ-casein zero calibrator without (red line) or with stirring of the solutions (blue line) during the primary (arrows 1 to 2) and secondary (arrow 3 to end) immunoreactions.

The chemical modification of the photonic dipstick chips’ surface with APTES was optimized with respect to the zero calibrator signal. The following treatments have been tested: 0.5 *v*/*v* aqueous APTES solution for 2 min, 2% *v*/*v* APTES for 2 and 20 min, or 2% *v*/*v* APTES solution in ethanol for 2, 20, and 60 min. In all cases, the photonic chips were spotted with bovine κ-casein at a concentration of 100 μg/mL and were first immersed for 5 min into a well containing rabbit anti-bovine κ-casein antiserum 100-times diluted with assay buffer and into a well containing the secondary antibody for another 5 min. As shown in Figure 3a, all conditions tested for chemical modification with aqueous APTES solution provided statistically the same zero calibrator signal values, whereas use of the APTES solution in absolute ethanol provided higher signal values compared to those received with aqueous solutions, especially when the incubation was prolonged to 20 min. Thus, chip surface modification with 2% *v*/*v* APTES in absolute ethanol for 20 min was adopted in the final protocol.

### 3.2. Assay Optimization

The photonic dipstick sensor allows the monitoring of reactions by immersion of the chip end with the MZI’s sensing windows directly into solutions and samples, abolishing the need for microfluidics and pumps. However, the implementation of flow increases the sensor’s response as it replenishes the reagents at the proximity of the transducer surface [35]. To compensate for the absence of flow, stirring was introduced by means of miniature magnetic stirrers in the microtiter wells used as reagents vials. In Figure 3b, the real-time responses obtained from chips coated with a 100 μg/mL bovine κ-casein solution and tested using 100-times diluted anti-bovine κ-casein antiserum both with and without stirring are presented. As shown, the signal increased approximately four times when stirring of the solutions into the wells was employed as compared to the signal obtained without stirring, due to acceleration of both primary and secondary immunoreactions, and this method was therefore adopted in the final protocol.

**Figure 4:**
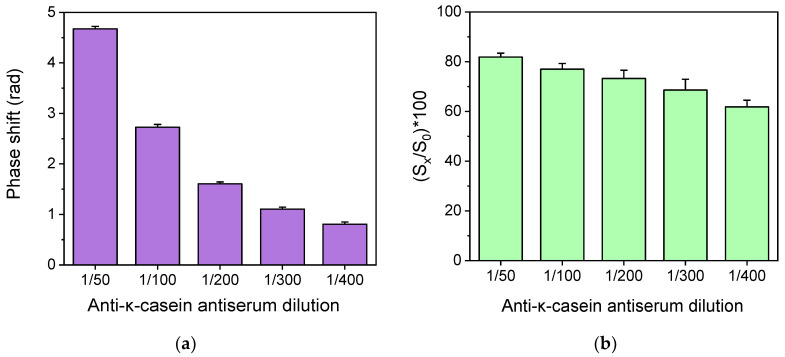
(**a**) Effect of rabbit polyclonal anti-bovine κ-casein antiserum dilution on the net zero calibrator signals obtained from chips spotted with a 100 μg/mL bovine κ-casein solution. Each column corresponds to the mean of 3 chips ± SD. (**b**) Effect of rabbit polyclonal anti-bovine κ-casein antiserum dilution on the percent signal value corresponding to a calibrator containing 0.5% *v*/*v* cow milk with respect to zero calibrator signals obtained from chips spotted with a 100 μg/mL bovine κ-casein solution. Each column corresponds to the mean of 3 chips ± SD.

The optimum concentration of κ-casein for spotting, as well as the anti-bovine κ-casein antiserum dilution, was determined with respect to zero calibrator signal values (maximum signal) and the assay’s sensitivity. To optimize the anti-bovine κ-casein antiserum dilution, the signals corresponding to calibrators containing 0 and 0.5% *v*/*v* cow milk in goat milk were determined for antiserum dilutions spanning from 1:50 to 1:400 using chips spotted with a 100 μg/mL κ-casein solution. Both calibrators were assayed after 100-times dilution with assay buffer. As shown in Figure 4a, the zero calibrator signals increased continuously as the antiserum dilution decreased and an adequate signal (signal > 1 rad) was achieved using anti-bovine κ-casein antiserum dilution equal to or lower than 200 times. At the same time, the assay sensitivity, as indicated by the percent signal value corresponding to a calibrator containing 0.5% *v*/*v* cow milk with respect to the zero calibrator (Figure 4b), increased as the antiserum dilution was also increased. Thus, in order to have an adequate zero calibrator signal and detection sensitivity, a 1:200 antiserum dilution was selected for the final protocol.

To define the optimum bovine κ-casein concentration for spotting, solutions with concentrations ranging from 20 to 200 μg/mL were tested with respect to the zero calibrator signal. As indicated in Figure S4, maximum plateau signal values were obtained when bovine κ-casein solutions with concentrations equal to or higher than 50 μg/mL were used for the spotting of the chips. Consequently, the concentration of 50 μg/mL was selected for further experimentation. A BSA solution with a concentration of 50 μg/mL was used for spotting of the reference sensor.

### 3.3. Matrix Effect

**Figure 5:**
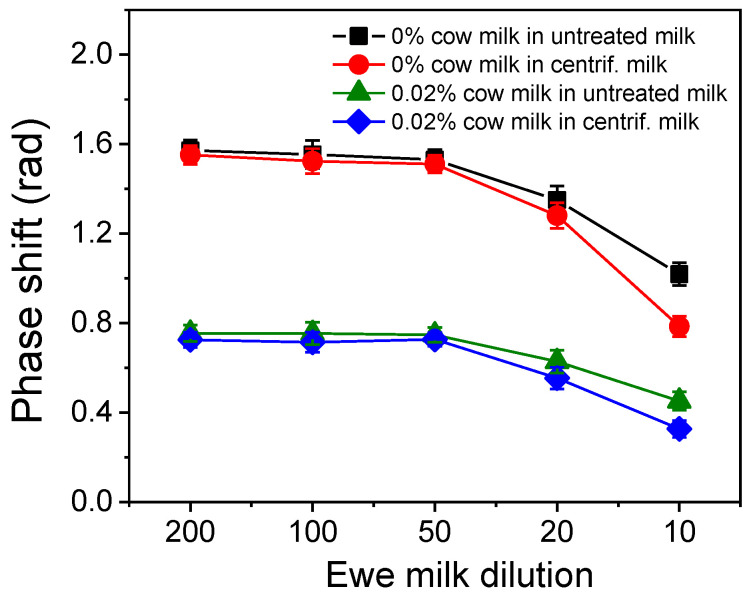
Net signal values versus milk sample dilution with assay buffer for calibrators containing 0 (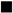, 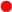) and 0.02 % *v*/*v* cow milk (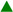, 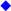) in centrifuged (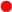, 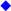) and untreated (non-centrifuged) ewe milk (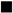, 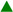). Each value corresponds to the mean of 3 measurements ± SD.

Milk is a food with high nutritional value since it contains hydrocarbons, lipids, and proteins, as well as several vitamins and minerals. Despite their high nutritional value, the same ingredients might interfere with the assay, affecting its performance. Lipids in particular could pose a problem due to their tendency to stick to surfaces. The most effective way to remove lipids is by centrifugation as they form a top layer through which the aqueous layer can be easily collected. Thus, ewe’s milk prior to and after centrifugation (15 min at 3000 g) was spiked with cow’s milk at concentrations ranging from 0.2 to 4.0% *v*/*v*, so that after 10- to 200-times dilution with the assay buffer, respectively, the percentage of cow’s milk in ewe’s milk was 0.02% *v*/*v* in all cases. As presented in Figure 5, reduced signals were obtained for 10- and 20-times milk dilutions compared to those obtained for milk dilutions equal to or higher than 50 times. This effect was more pronounced for the non-centrifuged milk, indicating some interference from the lipids. However, the effect in the net chip signal did not affect the assay sensitivity. Thus, in order to combine a high zero calibrator signal with detection sensitivity, a 50-times dilution of ewe milk was adopted in the final protocol.

Similar results were found for goat and donkey milk. As shown in Figure S5, the zero calibrator signal obtained for 50-times diluted ewe milk (1.66 rad) was similar to that obtained for the zero calibrator in 50-times diluted goat (1.59 rad) and donkey milk (1.69 rad). Furthermore, the percentage signal calculated for a calibrator containing 0.5% *v*/*v* cow milk in ewe, goat, or donkey milk with respect to the zero calibrator signal ranged from 54.8 to 58.5%.

### 3.4. Analytical Evaluation of the Developed Immunosensor

**Figure 6:**
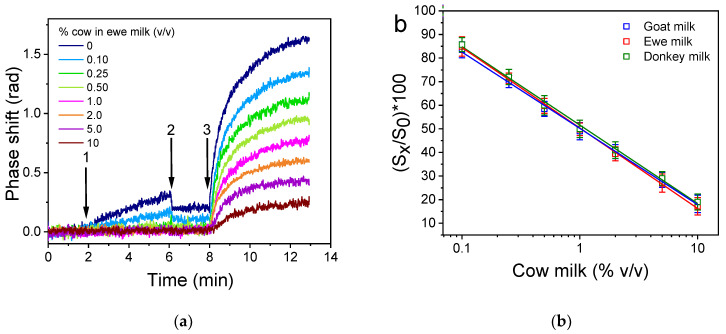
(**a**) Real-time signal responses obtained for cow milk calibrators in ewe milk. The arrows indicate the sequence of solutions in which the chip was immersed in: equilibration buffer (start to arrow 1), 1:1 *v*/*v* mixtures of calibrators prepared in ewe milk with the anti-bovine κ-casein antiserum (arrows 1 to 2), equilibration buffer (arrows 2 to 3), and anti-rabbit IgG antibody prepared in equilibration buffer (arrow 3 to end). (**b**) Typical calibration curves of cow milk in ewe (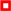), goat (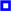), and donkey milk (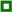). Each point is the mean value of 3 measurements ± SD.

The net chip responses obtained for cow milk calibrators in ewe (Figure 6a), goat, and donkey milk ranging from 0 to 10% (*v*/*v*) were used to prepare the respective calibration curves. As shown in Figure 6b, superimposed calibration curves were obtained for cow milk calibrators prepared in the milk of the other three species. The detection limit of the assay (LOD) was calculated as the concentration corresponding to the percent signal equal to 100-3SD of the mean zero calibrator signal of 10 measurements and was 0.05% *v*/*v* cow milk in ewe, goat, or donkey milk, with a linear working range from 0.1% to 10% *v*/*v*. The achieved limit of detection is 20 times lower than the maximum allowable limit set by the EU for milk adulteration (1%).

To evaluate the repeatability of the immunoassay, the intra- and inter-assay coefficients of variation (CVs) were determined. For this purpose, three different milk samples containing known amounts of cow milk in ewe, goat, and donkey milk were run in triplicate on the same day (intra-assay CV) and in duplicate at five random days during a period of 1 month (inter-assay CV). The intra- and inter-assay CVs determined were 4.0% and 6.5%, respectively, indicating the high repeatability of the measurements performed with the developed immunosensor.

The accuracy of the assay was determined by recovery experiments with samples of ewe, goat, and donkey milk spiked with cow milk at three different concentration levels (0.4%, 0.8%, and 2.5%). The % recovery was calculated according to the equation:

$$\%Recovery=\frac{\%\;cow\;milk\;determined}{\%\;cow\;milk\;added}\times 100$$

As presented in Table S1, the recovery values ranged from 92.0 to 105%, confirming the high accuracy of the proposed photonic dipstick immunosensor for the detection of milk adulteration.

### 3.5. Stability and Regeneration of the Immunosensor

The stability of the developed photonic dipstick immunosensor against repetitive immunoassay/regeneration cycles was also investigated. Thus, after the immunoreaction, the chip was immersed in 50 mM HCl solution for 2 min in order to remove the specific antibodies that were bound to the immobilized antigen, allowing the chip to be reused for another assay cycle. As shown in Figure S6a, chips spotted with bovine κ-casein could be regenerated up to 80 times, without any signal loss. After that, the signal was increased due to the increase of the residual signal, i.e., the signal due to incomplete specific antibody removal from the chip, which was determined by immersing the chip after regeneration in a secondary antibody solution (Figure S6b).

The stability of spotted chips that were stored at room temperature until use was also evaluated over a period of 28 days. It was found that spotted chips could be stored at RT for at least this time period without any signal decrease (Figure S7).

### 3.6. Comparison with Other Immunosensors

In Table 1, a comparison of various immunosensors developed for the detection of cow milk in dairy products from other species is presented. The first study regarding the detection of milk adulteration by an optical immunosensor was reported by Haasnoot et al. [32]. In this study, an SPR immunosensor capable of detecting milk adulteration at less than 0.1% (*v*/*v*) cow milk in milk from other species within 5 min was developed. The same team also developed an immunosensor for the determination of cow milk in goat and ewe milk based on a portable SPR instrument, exhibiting an LOD of 0.17% (*v*/*v*) cow milk for an assay of 7 min [33]. Similar levels of sensitivity have been reported for electrochemical immunosensors, which, however, required a total assay time of 30 to 60 min [29,30]. Highly sensitive determination of cow milk in ewe and goat cheese has been also reported using an immunosensor based on Sb_2_O_5_/SnO_2_ modified screen-printed electrodes [40]. The LOD achieved in this case was 0.07% (*v*/*v*). Adulteration of goat with cow milk was also investigated using a photonic immunosensor based on arrays of 10 integrated MZIs along with their respective broad-band light sources on a single chip, achieving a LOD of 0.04% in 25 min, including a 15 min preincubation step [34]. The same chip was applied to detect adulteration with cow milk in mozzarella and feta cheese, achieving a detection limit of 0.25 and 0.5% (*v*/*v*), respectively, for an assay duration of 9 min [36].

The proposed photonic dipstick immunosensor exhibits approximately the same detection sensitivity as the photonic immunosensor based on integrated MZIs and the electrochemical immunosensor based on Sb_2_O_5_/SnO_2_ modified screen-printed electrodes, with better sensitivity compared to all other immunosensors reported so far. In addition, the proposed immunosensor is fast, with a total assay time of 12 min, and there is no need for milk sample pretreatment. Furthermore, it is worth mentioning that this is the first time that donkey milk adulteration with cow milk was studied using an immunosensor. Although the detection sensitivity achieved with the proposed immunosensor is more than adequate for the specific application of ewe, goat, and donkey milk adulteration with cow milk, there is some space for further improvement. For example, an additional signal enhancement step could be employed by replacing the secondary antibody with a biotinylated one so as to use streptavidin for further signal enhancement. Thus, the bovine κ-casein-specific antibody concentration can be reduced, leading to an increase in competitive assay sensitivity. Of course, the implementation of an additional reaction step will increase the complexity and the duration of the whole assay procedure. However, if a higher detection sensitivity is crucial for a specific application, this approach will be applied. Another approach to increase the signal and detection sensitivity is to increase the sensing window’s length. To what extent this modification can improve assay performance must be proved experimentally.

## 4. Conclusions

In this study, a photonic dipstick immunosensor is used for the first time to detect ewe, goat, and donkey milk adulteration with cow milk. The proposed immunosensor could detect cow milk in milk from ewes, goats, and donkeys at concentrations as low as 0.05% (*v*/*v*) within 12 min. This limit of detection is far below the maximum acceptable limit for milk adulteration established by the European Union (EU), which is 1% *v*/*v*, whereas the linear dynamic range extends up to 10% *v*/*v* cow milk. The proposed photonic dipstick immunosensor does not require external pumps and microfluidics, thereby simplifying the instrumentation. In addition, no sample pretreatment, such as centrifugation of the milk samples, was needed, or preincubation of the mixture of calibrators with the antibody solution, further simplifying the assay procedure. The small size of the instrumentation, the excellent analytical characteristics achieved, and the simplicity of the developed method make the proposed sensor ideal for the on-site detection of milk adulteration.

## Figures and Tables

**Table 1:** Comparison of the proposed dipstick immunosensor with other immunosensors in the literature for determination of cow milk in various dairy products.

Immunosensor Type	Sample	Assay Time (min)	LOD (% *v*/*v* Cow Milk)	Working Range (% *v*/*v* Cow Milk)	Ref.
SPR	Ewe milk Goat milk	5	<0.1	0.1–10	[32]
Portable SPR	Ewe milk Goat milk	7	0.17	0.17–1.35	[33]
Amperometric	Ewe milk Goat milk Buffalo milk	30	0.1	not defined	[29]
Voltametric	Goat milk	60	0.01	0.1–100	[30]
QD-based voltametric	Ewe cheese Goat cheese	15	0.07	0.14–1.0	[40]
MZI	Ewe Goat milk	10	0.04	0.1–1.0	[34]
MZI	Mozzarella Feta cheese	~9	0.5 0.25	1.0–50 0.5–25	[36]
Photonic dipstick immunosensor	Ewe milk Goat milk Donkey milk	12	0.05	0.1–10	This work

## Data Availability

The relevant data are available by the corresponding author upon reasonable request.

## Supplementary Materials

The following supporting information can be downloaded at: https://www.mdpi.com/article/10.3390/s24175688/s1, Figure S1. Schematic of the process flow followed for the fabrication of the photonic silicon chips; Figure S2: Image of the optical set-up (left), portable instrument (middle) and photonic dipstick immunosensor chip connected to the optical adapter and immersed in the immunoreaction solution (right); Figure S3: Different snapshots of the platform software during chip immersion in: (a) assay buffer solution, (b) rabbit anti-bovine k-casein antiserum solution, and (c) anti-rabbit IgG solution. Top left panel: peak phase tracking in Fourier domain; Top middle panel: real-time evolution monitoring of the recorded spectra. The red spectrum is the one recorded at the start of each experiment, the blue is the temporal spectrum recorded every 150 ms, and the yellow one is the normalized spectrum obtained by dividing every recorded spectrum with a mean LED spectrum; Bottom left panel: real-time monitoring of the peak phases during chip equilibration with assay buffer (blue line: reference MZI; red line: working MZI); Right panel: software control buttons.; Figure S4. Effect of bovine κ-casein concentration in the spotting solution to the net zero calibrator signal values. Each column corresponds to the mean of 3 chips ± SD; Figure S5. Real-time signal responses obtained for the zero calibrator and a calibrator containing 0.5% *v*/*v* cow milk in (a) ewe, (b) goat, and (c) donkey milk. For the assay, all milk samples were diluted 50 times with assay buffer and then mixed at 1:1 volume ratio with a 200 times diluted rabbit anti-bovine κ-casein antiserum solution. The arrows indicate the sequence of solutions in which the chip was immersed to: 100 times diluted milk in assay buffer (start to arrow 1), mixtures of calibrators prepared in 50 times diluted milk with anti-bovine κ-casein antiserum (arrow 1 to 2), 100 times diluted milk in assay buffer (arrow 2 to 3), and anti-rabbit IgG antibody in 100 times diluted ewe milk (arrow 3 to end).; Table S1. Recovery of known amounts of % cow milk added in ewe, goat and donkey milk; Figure S6. (a) Zero calibrator signals obtained from a single chip after 85 regeneration cycles. Horizontal solid line corresponds to the mean value of the measurements, and the dashed lines to mean ± 2SD. (b) Zero calibrator (blue columns) and residual signals (pink columns) determined for the last 10 regeneration cycles (75–85) of the chip.; Figure S7. Zero calibrator signals obtained from chips stored at room temperature and assayed over a period of 28 days. Each column corresponds to the mean of 3 chips ± SD. Horizontal solid lines correspond to ±3SD.
