# Supplementary material for: Photonic Dipstick Immunosensor to Detect Adulteration of Ewe, Goat, and Donkey Milk with Cow Milk through Bovine κ-Casein Detection
Source: Sensors (Basel). 2024 Aug 31;24(17):5688. doi: 10.3390/s24175688 (PMC11398010; doi:10.3390/s24175688)
Supplement: Supplementary file 1 [file sensors-24-05688-s001.zip › sensors-3110092-supplementary.pdf]

Supplementary material

Photonic Dipstick Immunosensor to Detect Adulteration of Ewe, Goat, and Donkey Milk with Cow Milk through Bovine κ-Casein Detection

Dimitra Kourti [1,2], Michailia Angelopoulou [1], Eleni Makarona [3], Anastasios Economou [2], Panagiota Petrou [1], Konstantinos Misiakos [3] and Sotirios Kakabakos [1,*]

[1] Immunoassays–Immunosensors Lab, Institute of Nuclear & Radiological Sciences & Technology, Energy & Safety, NCSR "Demokritos", GR-15341 Aghia Paraskevi, Greece; d.kourti@rrp.demokritos.gr (D.K.); mi-kangel@ipta.demokritos.gr (M.A.); ypetrou@rrp.demokritos.gr (P.P.)
[2] Analytical Chemistry Lab, Department of Chemistry, National and Kapodistrian University of Athens, Panepistimiopolis Zografou, GR-15771 Athens, Greece; aeconomo@chem.uoa.gr
[3] Institute of Nanoscience & Nanotechnology, NCSR "Demokritos", GR-15341 Aghia Paraskevi, Greece; e.makarona@inn.demokritos.gr (E.M.); k.misiakos@inn.demokritos.gr (K.M.)
* Correspondence: skakab@rrp.demokritos.gr

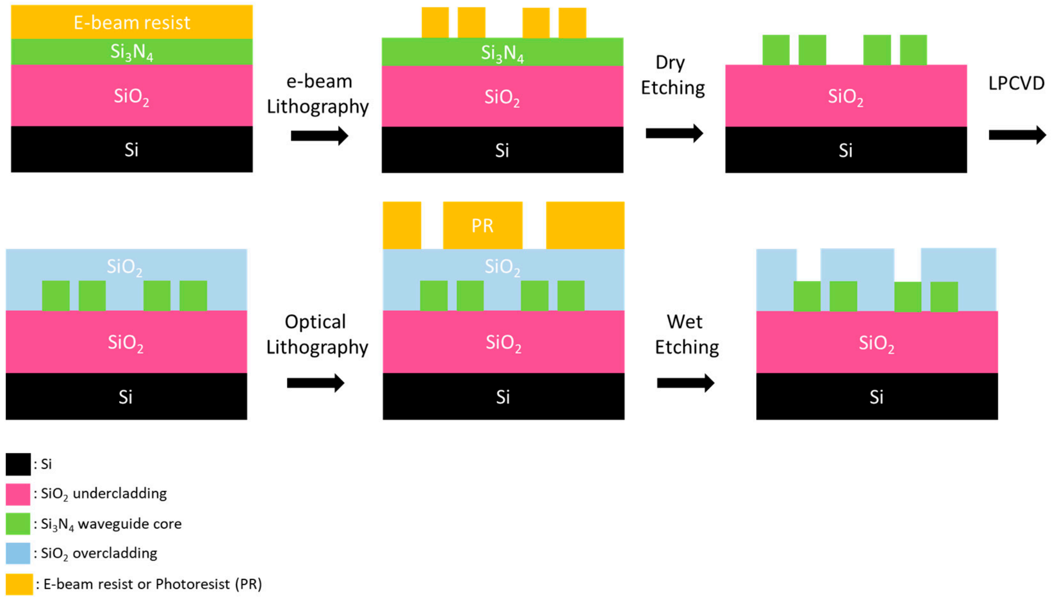

Figure S1. Schematic of the process flow followed for the fabrication of the photonic silicon chips.

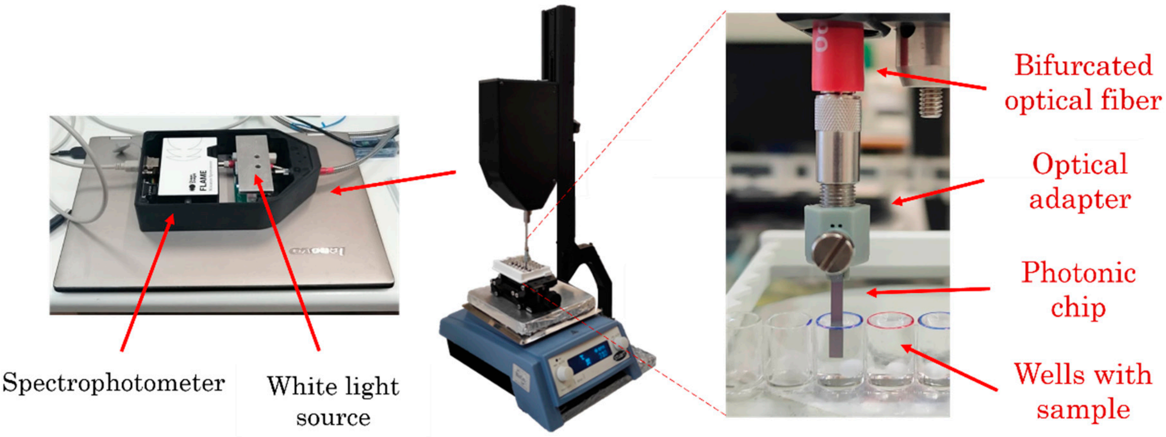

Figure S2. Image of the optical set-up (left), portable instrument (middle), and photonic dipstick immunosensor chip connected to the optical adapter and immersed in the immunoreaction solution (right).

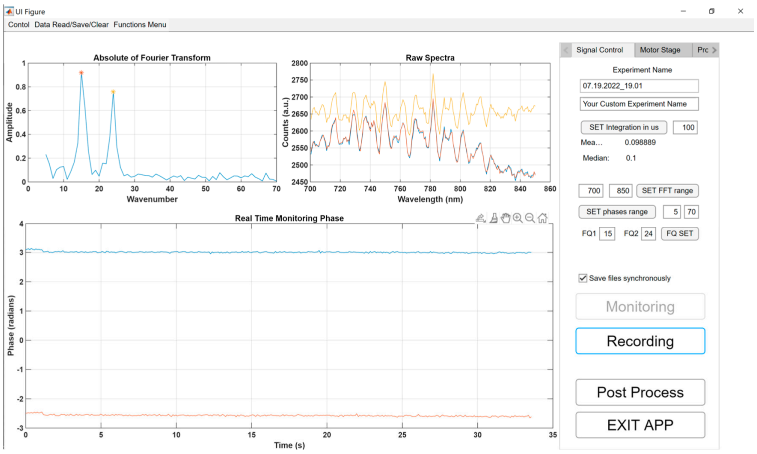

(**a**)

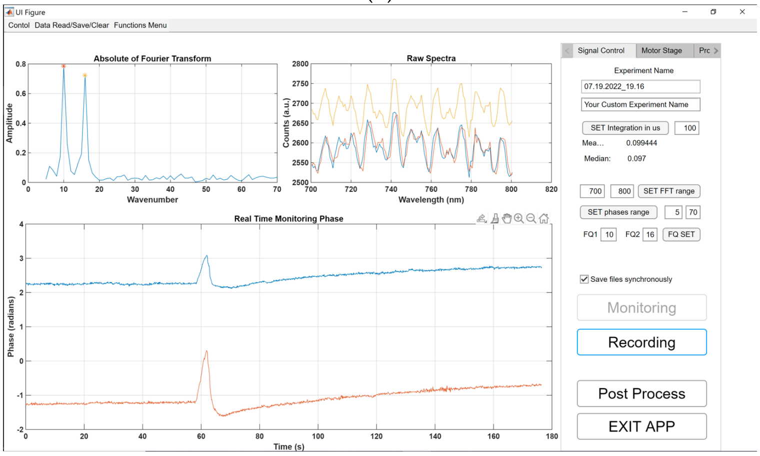

(**b**)

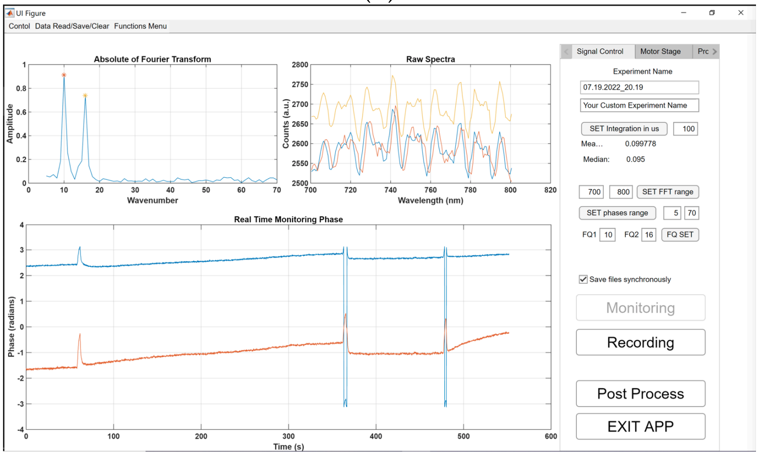

(**c**)

Figure S3. Different snapshots of the platform software during chip immersion in: (**a**) assay buffer solution, (**b**) rabbit anti-bovine k-casein antiserum solution, and (**c**) anti-rabbit IgG solution. Top left panel: peak phase tracking in the Fourier domain; Top middle panel: real-time evolution monitoring of the recorded spectra. The red spectrum is the one recorded at the start of each experiment, the blue is the temporal spectrum recorded every 150 ms, and the yellow one is the normalized spectrum obtained by dividing every recorded spectrum with a mean LED spectrum; Bottom left panel: real-time monitoring of the peak phases during chip equilibration with the assay buffer (blue line: reference MZI; red line: working MZI); Right panel: software control buttons. The peaks observed in the signal of both the working (blue line) and the reference sensors (red line) at the time points where the chip is moved from a reagent well to another are due to refractive index changes caused by brief exposure to air. These "peaks" are smoothed out when the net chip signal is calculated following the Equation mentioned in Section 2.5.

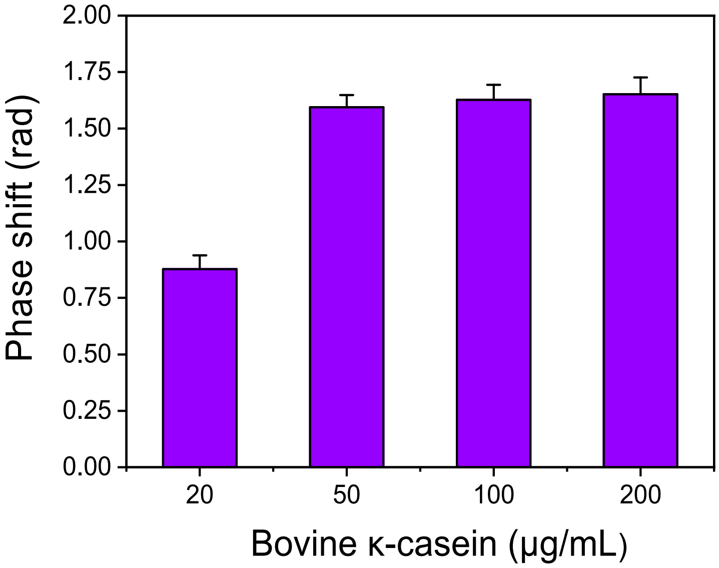

Figure S4. Effect of bovine κ-casein concentration in the spotting solution to the net zero calibrator signal values. Each column corresponds to the mean of 3 chips ± SD.

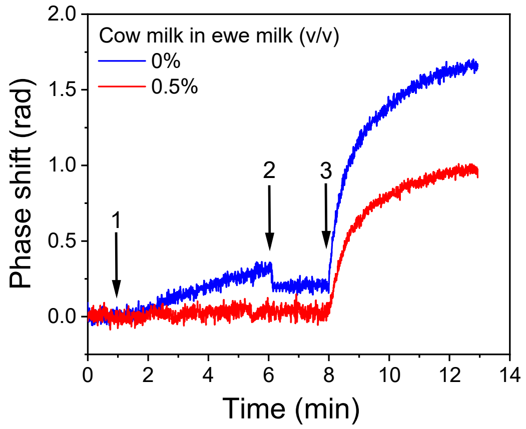

(**a**)

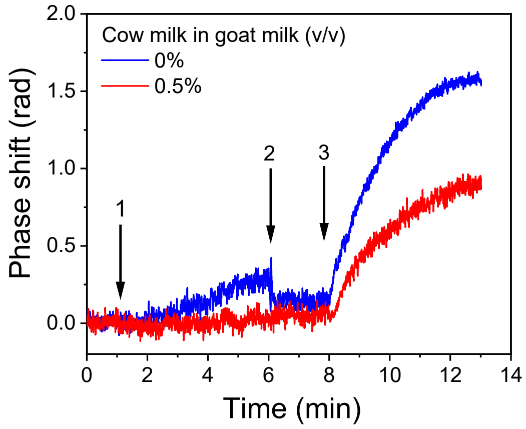

(**b**)

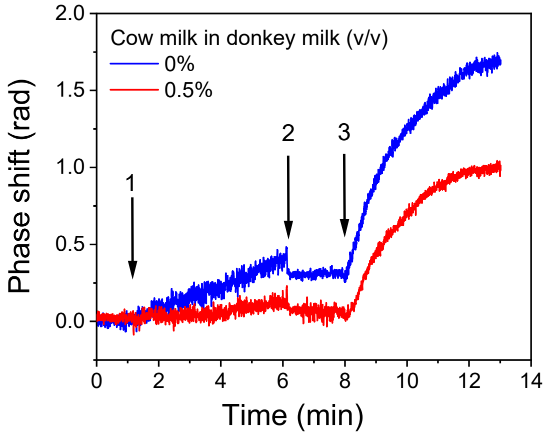

(**c**)

Figure S5. Real-time signal responses obtained for the zero calibrator and a calibrator containing 0.5% v/v cow milk in (**a**) ewe, (**b**) goat, and (**c**) donkey milk. For the assay, all milk samples were diluted 50 times with assay buffer and then mixed at a 1:1 volume ratio with a 200-times diluted rabbit anti-bovine κ-casein antiserum solution. The arrows indicate the sequence of solutions in which the chip was immersed to: 100-times diluted milk in assay buffer (start to arrow 1), mixtures of calibrators prepared in 50-times diluted milk with anti-bovine κ-casein antiserum (arrows 1 to 2), 100-times diluted milk in assay buffer (arrows 2 to 3), and anti-rabbit IgG antibody in 100-times diluted ewe milk (arrow 3 to end).

Table S1. Recovery of known amounts of % cow milk added in ewe, goat, and donkey milk.

Sample	Amount added (% v/v cow milk)	Amount determined (% v/v cow milk)	Recovery %
Ewe milk	0.40	0.39	97.5
	0.80	0.77	96.2
	2.5	2.6	104
Goat milk	0.40	0.42	105
	0.80	0.74	92.5
	2.5	2.4	96.0
Donkey milk	0.40	0.41	102
	0.80	0.79	98.7
	2.5	2.3	92.0

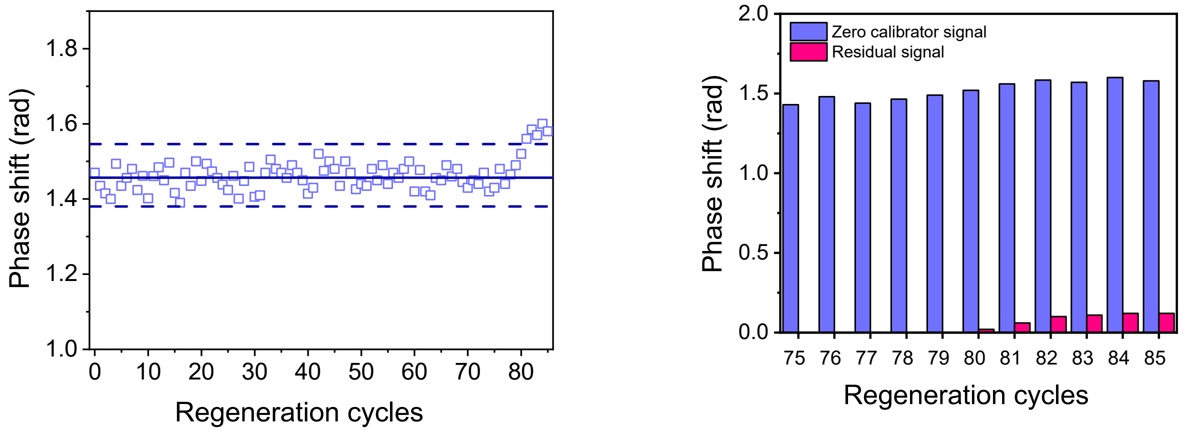

(**a**) (**b**)

Figure S6. (**a**) Zero calibrator signals obtained from a single chip after 85 regeneration cycles. Horizontal solid line corresponds to the mean value of the measurements, and the dashed lines to mean ± 2SD. (**b**) Zero calibrator (blue columns) and residual signals (pink columns) determined for the last 10 regeneration cycles (75–85) of the chip.

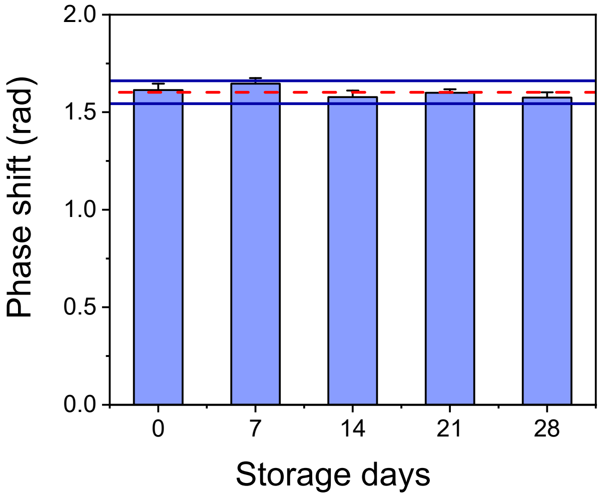

Figure S7. Zero calibrator signals obtained from chips stored at room temperature and assayed over a period of 28 days. Each column corresponds to the mean of 3 chips ± SD. Horizontal solid lines correspond to ± 3SD.
